# Defining matrix Gla protein expression in the Dunkin-Hartley guinea pig model of spontaneous osteoarthritis

**DOI:** 10.1186/s12891-021-04735-2

**Published:** 2021-10-12

**Authors:** Xun Ma, Zhan Zhang, Xinyuan Kang, Chunbo Deng, Yingwei Sun, Yanjun Li, Desheng Huang, Xueyong Liu

**Affiliations:** 1 Department of Rehabilitation, Shengjing Hospital of China Medical University, No.16, Puhe Street, Shenyang North New Area, Shenyang, 110134 Liaoning Province China; 2 Department of Orthopaedics, Shengjing Hospital of China Medical University, Shenyang, Liaoning Province China; 3 Department of Orthopedics, Central Hospital of Shenyang Medical College, Shenyang, Liaoning Province China; 4 Department of Radiology, Affiliated Hospital of Liaoning University of Traditional Chinese Medicine, Shenyang, Liaoning Province China; 5 Department of Radiology, Shengjing Hospital of China Medical University, Shenyang, Liaoning Province China; 6 Department of Mathematics, College of Basic Medical Sciences, China Medical University, Shenyang, China

**Keywords:** Matrix Gla protein, Osteoarthritis, Guinea pig, Proteomics

## Abstract

**Background:**

Matrix Gla (γ-carboxyglutamate) protein (MGP) is considered a strong inhibitor of ectopic calcification, and it has been associated with OA severity, although not conclusively. We utilized male Dunkin-Hartley (DH) guinea pigs to investigate the expression of MGP throughout aging and disease pathogenesis in a spontaneous model.

**Method:**

Twenty-five male DH guinea pigs were obtained and nurtured to several timepoints, and then randomly and equally divided by age into five subgroups (1-, 3-, 6-, 9-, and 12-months, with the 1-month group as the reference group). DH guinea pigs in each group were euthanized at the designated month-age and the left or right medial tibial plateaus cartilages were randomly excised. OA severity was described by modified Mankin Score (MMS) at microscopy (Safranin O/Fast Green stain). Proteomic evaluation using isobaric tags for relative and absolute quantification (iTRAQ) was performed to validate the age-related changes in the MGP profiles, and immunohistochemistry (IHC) methods were applied for semi-quantitative determination of MGP expression in articular cartilage.

**Results:**

The histopathologic findings validated the increasing severity of cartilage degeneration with age in the DH guinea pigs. The MMS showed significant, stepwise (every adjacent comparison *P* < 0.05) disease progression with month-age. The iTRAQ indicated that MGP levels increased significantly with advancing age (P < 0.05), as supported by the IHC result (P < 0.05).

**Conclusion:**

Increased expression of MGP in male DH guinea pigs was present throughout aging and disease progression and may be link to increased OA severity. Further studies are needed to investigate and confirm the association between MGP levels and OA severity.

**Supplementary Information:**

The online version contains supplementary material available at 10.1186/s12891-021-04735-2.

## Introduction

Osteoarthritis (OA) is the most common degenerative joint disease of the middle-aged and elderly [1]. OA may lead to reduced quality of life, and it is a major cause of work incapability and disability. OA is an important medical challenge across the globe [2], but the etiology, risk factors, and pathophysiology remain unclear. At present, early detection and treatment of OA are important. Identifying new therapeutic targets and biomarkers of OA is a research focus, but biomarkers of OA severity are rarely reported.

Matrix Gla (γ-carboxy glutamate) protein (MGP) is a vitamin K-dependent protein that was first isolated from bovine bone [3]. Uncarboxylated (inactive) MGP is converted into carboxylated (active) MGP by vitamin K, and by this way it is possible to detect the vitamin K status in vivo [4]. MGP may function as an inhibitor of ectopic calcification in cardiovascular tissues and cartilage, but the detailed mechanism remains unclear. MGP has been shown to inhibit BMP-2-induced calcification and spontaneous vascular calcification [5], and MGP mutation may lead to Keutel syndrome, a genetic disorder that is mainly characterized by multiple pathological changes including cartilage calcification, brachydactyly, and pulmonary artery stenosis [6]. Meanwhile, MGP was reported as an anti-inflammation cytokine, may have the effects of alleviating inflammatory reaction in patients with acute pancreatitis and arthritis [7, 8]. rs1800802 located in an MGP polymorphic site has been reported in association with an increased OA risk in a Han Chinese population [9, 10]. However, although a potential association between MGP and OA has also been suggested, there is still no consensus or detailed mechanism regarding the presence and effect of MGP in OA severity and natural history.

The Dunkin-Hartley (DH) guinea pig is considered a useful and common animal model of spontaneous OA, with several features that are similar to human OA pathogenesis and progression [11, 12]. Based on previous studies [13, 14], MGP is hypothesized to be associated with OA pathogenesis and/or prognosis. Thus, this study was conducted to investigate MGP levels in a naturally-occurring model at key points throughout aging.

## Materials and methods

### Animal experiments

All animal experiments were performed with approval of the Animal Ethics Committee of Shengjing Hospital of China Medical University (No. 2016PS284K), which complies with the Guide for the Care and Use of Laboratory Animals, 8th edition, published by the United States National Institutes of Health (NIH Publication, 2011) [15] and Animal Research: Reporting of In Vivo Experiments (ARRIVE) Guidelines 2.0 [16]. The ARRIVE checklist was available in Supplementary Materials.

A total of 25 male DH guinea pigs with healthy appearance, appetite and activity were obtained from Beijing Huafukang Bioscience Co. Inc. Each month (1-, 3-, 6-, 9-, and 12-months), 5 guinea pigs were randomly selected as the marked month-age group by random number table, with the 1-month-old guinea pigs as the reference group, other month-olds as observation groups. The animals were nurtured in the Animal Department of Shengjing Hospital Affiliated to China Medical University. Every random two guinea pigs in each cage were kept and drank freely. The indoor temperature was controlled at 20°C–25°C and the humidity was kept at 40–50%. The DH guinea pigs were conventional feeding with standard guinea pig chow (purchased from Shenyang Maohua Biotechnology Co., Ltd), and for the need of vitamin C, they were fed with fresh vitamin C-rich cabbage every day for the first week and 2–4 times for the rest weeks.

All the experimental procedures were performed in the Experimental Center of Shengjing Hospital of China Medical University. During the entire study period, the investigators in charge of animal raising were blind for all the subsequent operations involving animals, and the investigators performed the experiment were not aware of the grouping. When the groups reached the target age, the animals were weighed. The guinea pigs were then placed under general anesthesia by intraperitoneal injection of 3% pentobarbital sodium, followed by intact knee specimens obtained, and then guinea pigs were then euthanized by intracardiac air injection embolization. For every guinea pig in a certain group, a randomly determined left or right-side knee specimen was selected for proteomics analysis, and the other side knee were used for the immunohistochemistry (IHC) examination in animal experiments; and, cartilages of medial tibial plateau are used in both of IHC and proteomics procedures. The gross specimens were photographed with a Canon 5D Mark3 camera.

### Proteomics analysis

After gross knee specimens were collected from all groups, the knee specimens were immediately put into a 4°C incubator and randomly taken photographs, temporary preserved in liquid nitrogen and quickly transferred to a − 80 °C refrigerator for long-term preservation. Cartilages of medial tibial plateau obtained from a random lower limb of every guinea pig were sampled under liquid nitrogen environment, and interested cartilages of each group were mixed together for the proteomic analyses. The total protein of the interested cartilages of each group was extracted by a protein extraction kit. Isobaric tags for relative and absolute quantification (iTRAQ) tests were performed in triplicate in each group, and stepwise comparisons of differentially expressed proteins (DEPs) were made between sequential groups to identify proteins with change of 1.2-fold or greater (*P* < 0.05).

### Histopathologic analysis

Total knee specimens without cartilage extraction were stored in 4% paraformaldehyde for one week, then the specimen was washed with running water for 12 h, and then decalcified in EDTA decalcification solution. The decalcification was at 25 °C and the durations depended on the size of the specimen. The decalcification time of knee joint specimens of 1-month-old guinea pigs was about 4 weeks, and that of 12-month-old guinea pigs was 12 weeks. The rest month-olds were between 4 and 12 weeks. After complete decalcification, the gross knee joint was sectioned from the medial collateral ligament along the coronal plane. The wax-blocking procedure is as follows: 1. Wash with running water for 8 h, 75% alcohol overnight, 85% alcohol for 3 h, 95% alcohol (I) for 3 h, 95% alcohol (II) for 2 h, 100% alcohol (I) for 2 h, 100% alcohol (II) for 2 h, xylene (I) for 30 min, xylene (II) for 30 min, paraffin (I) for 60 min, paraffin (II) for 90 min. Then the wax block was made by tissue embedding machine. The embedded wax blocks were sliced with a paraffin section machine with a thickness of 3 μm. Pick up the chips, bake them in a 60 °C toaster, and bake them in a 60 °C oven overnight.

Then Safranine O-fast green staining was performed. Medial tibial plateau area was chosen for histopathologic analysis due to reported as the earliest-phase and most common degeneration region in knee OA. The severity of OA was graded according to a modified Mankin score (MMS) [17, 18]. The MMS comprised four sections: Structure, Cellularity, Matrix staining, Tidemark integrity. Correlation analysis is used for the relationship between MMS and IOD/area. All sections were estimated blindly by an observer, triplicate values were performed for MMS.

### Immunohistochemistry (IHC)

Coronal sections of the knee were prepared and used for the IHC analysis, and medial tibial plateau area was used due to the statements mentioned above. For the antigen retrieval process, the paraffin section was placed in sodium citrate buffer (pH 7.2), heated with high fire level in microwave oven for 7 min, then cooled to room temperature for subsequent operation. After determining antigen and blocking endogenous peroxidase activity, obtained sections were incubated overnight with primary antibodies (anti-MGP, Sigma, SAB2101477, 1:50) at 4 °C. A secondary antibody with peroxidase-linkage was then added for 30 min at room temperature. 3,3′-diaminobenzidine tetrahydrochloride (DAB) was applied to detect staining. The sections were then immediately dehydrated with progressively ascending concentrations of ethanol solutions, cleared with xylene, and mounted with a coverslip of DePeX medium. IPP (version 6.0) image analysis software was used for semi-quantitative IHC analysis of MGP expression by using the average optical density (OD) (IOD/sum) value to indicate the depth of staining of MGP (+) particles in the chondrocytes, with deeper browns indicating higher levels of MGP expression. Negative control was conducted with only secondary antibody utilized, and 1-month group was used as reference group.

### Statistical analysis

All results are expressed as means ± standard deviation (SD). SPSS version 23.0 (IBM, Rochester, MN, USA) was used for all statistical analyses. Differences between means were analyzed by one-way analysis of variance (ANOVA) when time was the independent variable and applied data were variance homogeneous and conformed normal distribution. Correlation analysis was used for the relationship between MMS and IOD/area.

Inter- and intra-observer agreement were measured with the intraclass correlation coefficient (ICC) [19], single measurements, and absolute agreement. Generally, ICC 0.70 is set as a minimum standard for test-retest reliability, with ICC < 0.40 indicating weak correlation, 0.41 to 0.60 moderate correlation, 0.61 to 0.80 substantial correlation, and > 0.81 describing near-perfect agreement. Statistical significance was set at *P* < 0.05 for all analyses.

## Results

### Characteristics of the guinea pigs

A total of 25 DH guinea pigs were included in this study. From baseline to specimen collection, all of the guinea pigs were in healthy appetite and activity, and no unplanned death occurred. The weight changes of the guinea pigs from 1-month to 12-months are shown in Fig. 2 and Table 2.

### Histopathologic analysis

**Fig. 1:**
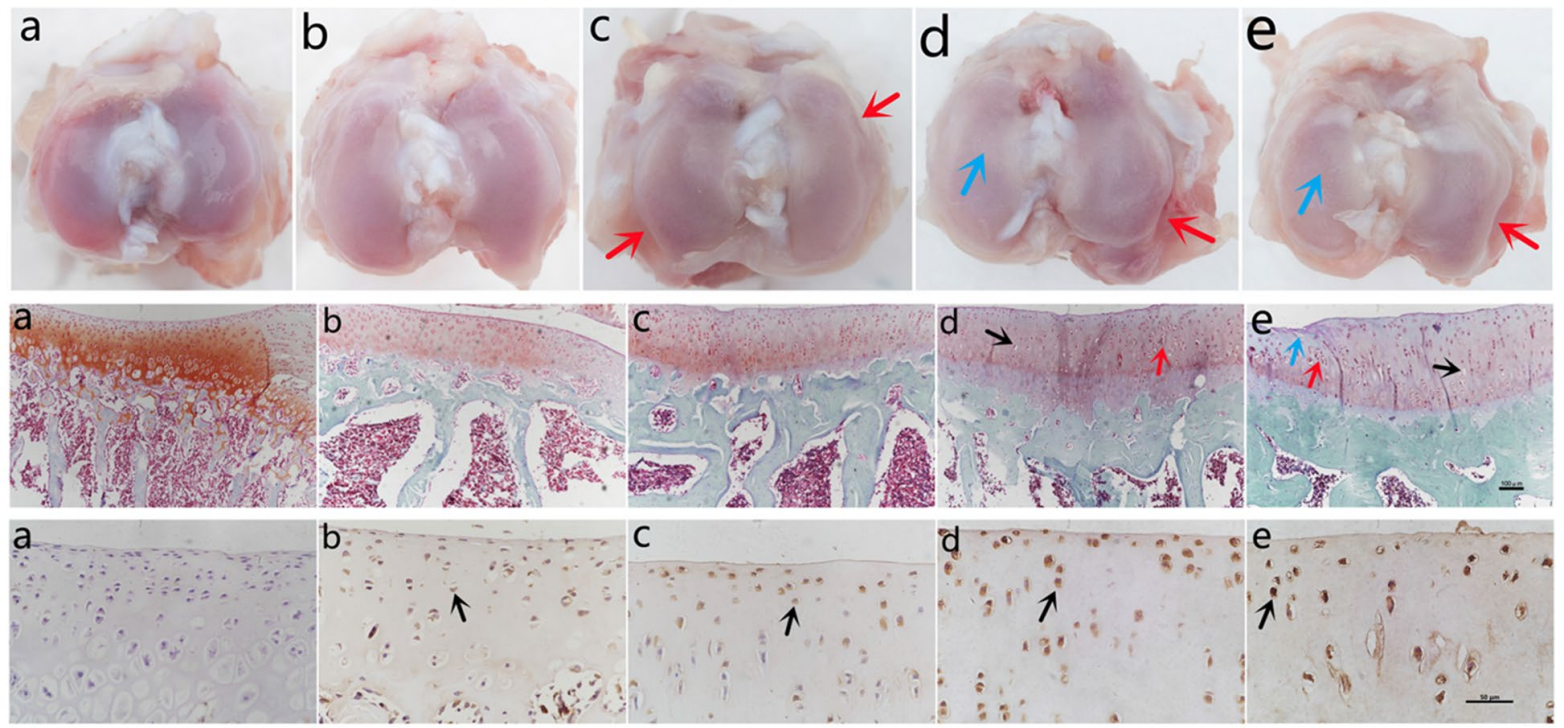
Histopathological progression of spontaneous osteoarthritis with aging in the DH guinea pig model. Row 1 (a) to (e) shows the gross histopathological changes in the knee joint from 1-month to 12-months. Red arrow designated rough surface of knee cartilage, and orange arrows indicated degenerative area. Row 2 (a) to (e) shows the microscopic changes with aging in safranine O-fast green-stained medial tibial plateau cartilages (respectively 1-, 3-, 6-, 9- and 12-month-old). Black arrows designated loss of chondrocytes in lacunas, purple arrows indicated chondrocyte cluster, and green arrows meant multiple cracks of cartilage surface. Row 3 (a) to (e) shows the progressive increase in MGP levels detected by immunohistochemistry, from 1-month to 12-months in medial tibial plateau cartilages. Yellow arrows designate positive MGP-expressed chondrocytes

Macroscopically, the articular surface was smooth and intact in the reference group, and a small amount of colorless and transparent synovial fluid could be seen in the articular cavity. As the guinea pigs aged, the articular fluid gradually became turbid and yellow and the articular surface showed gradual degeneration with cartilage slightly depressed. In the oldest group (12-months), the articular cartilage was rough and dull, with a thinner cartilage matrix and more cartilage depression, and osteophyte formation was visible at the edges of the joint. (Fig. 1 and Table 1), high quality figures with size marker were provided in Supplementary Materials.

**Table 1:** Histological manifestations of osteoarthritis by age (in months) in Dunkin-Hartley guinea pigs

Age (months)	Whole specimen	Microscopic staining (Medial tibial plateau cartilage)	IHC*
1	Articular surface was smooth and intact, and a small amount of colorless and transparent synovial fluid was seen in the articular cavity. No osteophyte was seen.	No pathologic change. Evenly distributed staining, morphology, and staining of cartilage matrix and chondrocytes.	No MGP expression or only a few light-stained cells.
3	With increasing age, the synovial fluid became turbid and yellow, the articular surface degenerated progressively like getting rougher, duller and thinner, and small areas of cartilage were slightly depressed; No obvious osteophyte was seen.	Tiny cracks were seen in cartilage surface, cell aggregation and cloning were occasionally-seen.	MGP was expressed in a small area of chondrocytes in the superficial layer of the joint with light staining, and with few positive-stained cells.
6		Damaged cartilage surface and cartilage layer structure with significant decreases in cartilage matrix staining and number of chondrocytes as well as more cell clusters and clones.	MGP positive chondrocytes were found in the deep layer of the cartilage.
9		Rough articular surface, disordered structure and irregular arrangement of chondrocytes, necrosis of chondrocytes, and empty cartilage sac.	The whole layer of chondrocytes was significantly positive, and the staining was significantly deeper than in the 6-month-old group.
12	Synovial fluid was obviously reduced, yellow and thick. Rough and dull articular cartilage surface, thin cartilage matrix, many cartilage depressions and losses, and sclerosing osteophytes were obviously seen.	Severely damaged cartilage structure and reduced number of chondrocytes with empty cartilage vesicles; staining of matrix was lost.	The positive cartilage staining was further deepened compared to the 9-month-old group.

*IHC = Immunohistochemistry. The cytoplasm of MGP-positive chondrocytes in articular cartilage were stained brown. MGP = Matrix Gla (γ-carboxyglutamate) protein

**Fig. 2:**
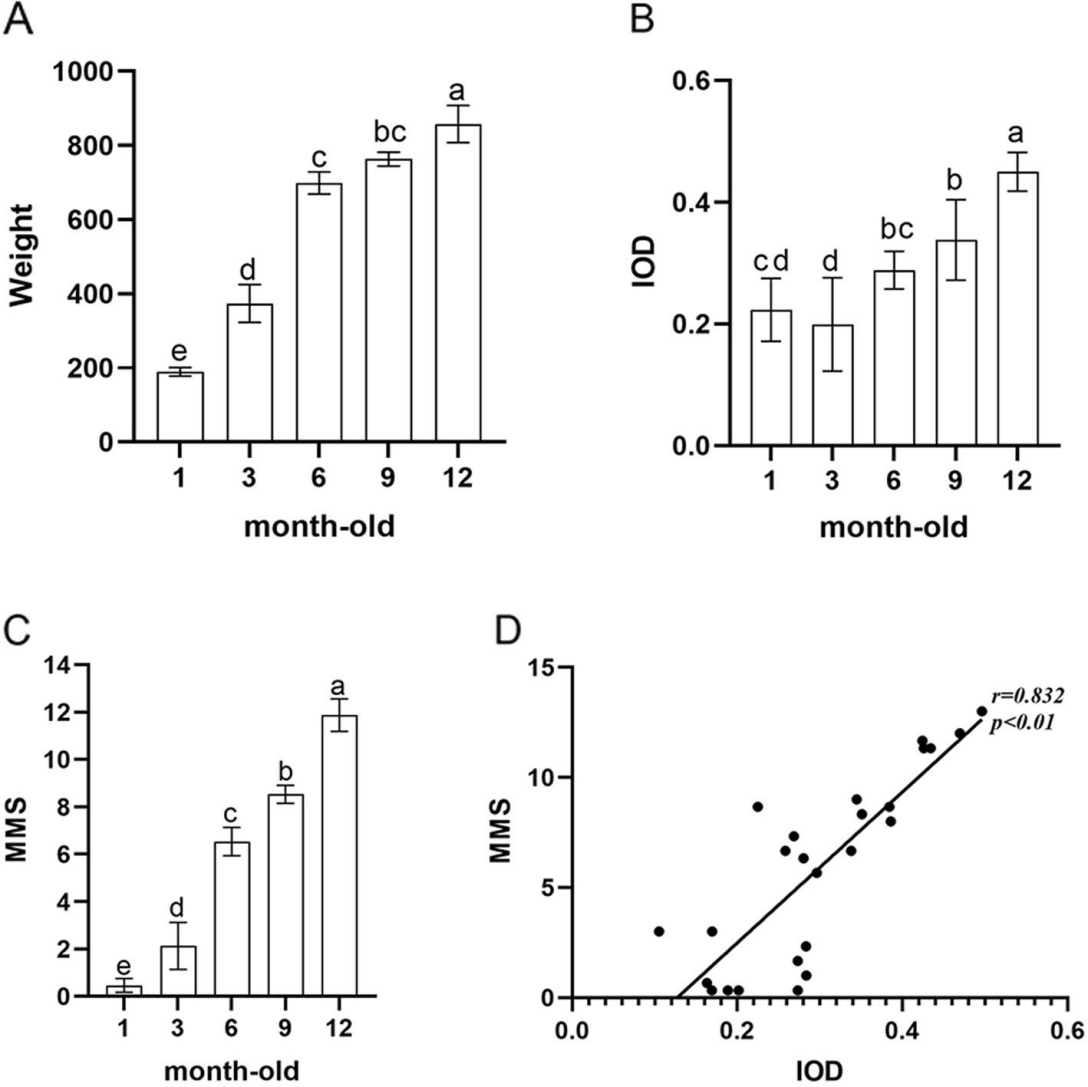
Comparison of changes across month-age groups in the DH guinea pig spontaneous osteoarthritis model.** A** Weight changes with aging. The weight of the DH guinea pigs shows a generally escalating trend with increasing month-age. The month-to-month change is statistically significant where noted. **B** Modified Mankin Scores (MMS) of knee cartilages in every group. MMS tests show significant month-by-month increases with age in the DH guinea pigs. **C** In IHC analysis, MGP IOD values in every month-age. There is also a general trend of monthly increase of MGP expression in the joint in the aging DH guinea pigs. **D** The correlation analysis of MMS and MGP IOD of IHC analysis. The analysis confirms a significant positive correlation between MGP expression and OA severity (as MMS). *The same letter between different groups represented no significant difference

At microscopical sights, no pathological manifestations of OA were observed in the reference group. For the cartilage structures of medial tibial plateau cartilage, tissue sections from the reference group showed non-degenerated cartilage structure, chondrocytes, and staining of the matrix, and no manifestations of degenerative disease were observed. At 3-months, the articular surface showed only tiny cracks with occasional cell aggregation and cloning, and no cell morphology or matrix abnormalities were seen. At 6-months to 9-months, progressive damage was indicated by roughened cartilage surface and irregular cartilage layer structure, the number of chondrocytes was significantly decreased, and necrosis and cell clusters and clones were gradually increased. By 12 months of age, the cartilage structure of knee was severely damaged and the number of chondrocytes was significantly decreased. In addition, the number of empty cartilage vesicles was increased, and the staining of the matrix was generally lost. (Fig. 1 and Table 1). These histopathological manifestations were in accordance with previous studies [20, 21]. The histopathological changes were analyzed semi-quantitatively by MMS (Fig. 2 and Table 2), these were continuous data obtained after scoring conform to the normal distribution and conformed normal distribution. The score increased continuously from 1-month (reference group) to 12-months, and the increase was significant between every adjacent group (*P* < 0.05).

**Table 2:** Weights, outcomes of MMS and iTRAQ analysis of every month-age group

Age (months)	Weight (g, Mean ± SD)	MMS (Mean ± SD)	FC1*	P1*	FC2	P2	FC3	P3
1	189.50 ± 11.84	Reference						
3	373.90 ± 50.80	2.13 ± 0.99	1.283	0.4750	1.039	0.9070	1.021	0.9087
6	698.80 ± 29.96	6.53 ± 0.60	1.385	0.2831	1.287	0.0668	1.183	0.0592
9	763.60 ± 18.71	8.53 ± 0.38	2.277	0.0437	2.033	0.0022	1.678	0.0045
12	857.90 ± 50.05	11.87 ± 0.69	1.806	0.0259	1.205	0.0449	1.042	0.4457

*FCs and Ps represented fold changes and *P*-values of every round of three-time iTRAQ

### Proteomics

Proteomics analysis was performed by three-time repeat iTRAQ to detect DEPs over time. Respectively 1221, 1413, and 1400 proteins were screened in the three iTRAQ runs, and the results confirmed a general increase in MGP expression over time, significantly 1.2-fold changes of MGP levels were detected in two of three times (Tables 2 and 3).

**Table 3:** Original and transformed data of IOD

Method	Month				
	1	3	6	9	12
Original data					
IOD-Aver	0.223	0.199	0.288	0.338	0.45
iTRAQ-G1*	1	1.283	1.385	2.277	1.806
iTRAQ-G2	1	1.039	1.287	2.033	1.205
iTRAQ-G3	1	1.021	1.183	1.678	1.042
Transformed data					
IOD-Trans	1	0.916	1.443	1.520	2.182
iTRAQ-G1	1	1.283	1.385	2.277	1.806
iTRAQ-G2	1	1.039	1.287	2.033	1.205
iTRAQ-G3	1	1.021	1.183	1.678	1.042

*iTRAQ G1 to G3 presented round 1 to round 3

### Immunohistochemistry

The cytoplasm of MGP-positive chondrocytes in knee cartilage are stained brown. Based on this, there was no MGP expression in reference group chondrocytes. The chondrocytes in the 3-month group showed slight MGP positivity over a small area of cartilage surface. A deeper layer and larger area of MGP (+) chondrocytes was observed in the 6-month group, and in the 9-month to 12-month groups, MGP (+) chondrocytes were detected over the entire area of the cartilage selection, and went progressively deeper (Figs. 1 and 2). The IHC sections were independently analyzed by two independent investigators, and the two sets of results were in accordance and deemed credible (ICC = 0.809, *P* < 0.001).

To compare the mean IOD values (IHC) and the iTRAQ results, the Taylor series expansion method was used to calculate the expected values of the ratio of the data measured at each timepoint to the data measured at the first timepoint, and then the IOD data were proportionally converted according to the following derivation formula:

$$\left(\frac{x_n}{x_1}\right)=\frac{m_n}{m_1}+\frac{m_n}{m_1^3}D\left({x}_1\right)-\frac{1}{m_1^2} Cov\left({x}_1,{x}_n\right)$$

m_1_: is the mean of the first measurement time point and M_n_ is that of the nth timepoint;
x_1_: is the observed value at the first measurement time point, x_n_ is that of the nth time point;
D (x_n_): is the variance of the nth measurement point;
CoV (x_1_, x_n_): is the covariance of x_1_, x_n_.

**Fig. 3:**
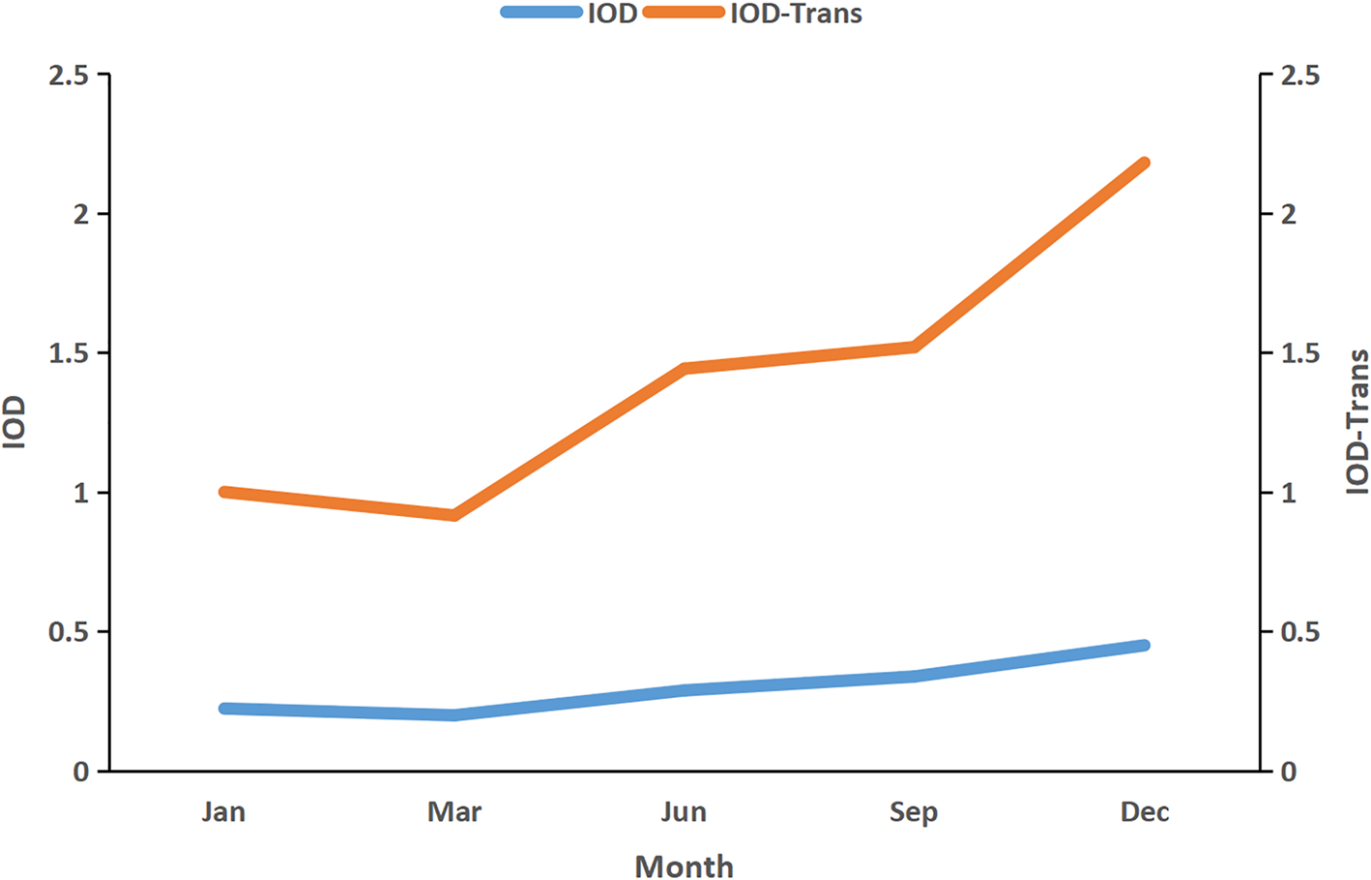
Correlation between IOD and IOD-trans transformed from iTRAQ values. The correlation analysis results showed that the correlation coefficients of IOD data and IOD-trans obtained from iTRAQ data were greater than 0.6. Due to the small sample size, the correlation between IOD data and iTRAQ groups was not significant

The converted data from each group were used to check for correlation between the proportional change of the mean IOD values and the change trend of the iTRAQ data (Table 3). The correlation analysis confirmed a strong positive association (ICC > 0.6) between the IOD measurements and the iTRAQ data trends (Fig. 3 and Supplementary Table 1). However, due to the small sample size, this finding did not reach significance.

## Discussion

Besides potential strongly inhibiting ectopic calcifications in cartilage and cardiovascular tissues reported [22], MGP is reported to alleviate inflammation in OA [23]. MGP has been identified as a biomarker of OA [22], and a correlation between MGP and severity of degeneration of the nucleus pulposus has also been reported [24]. However, data regarding the link between MGP expression in articular cartilage and OA incidence and severity have been regarded as inconclusive. The results of the present study show that increasing MGP expression is significantly associated with aging and spontaneous progression of OA in this animal model; this is consistent with serum MGP findings in humans with OA [22].

MGP is a vitamin K-dependent γ-carboxylated protein that has four known forms: completely carboxylated (cMGP), uncarboxylated (ucMGP), phosphorylated (pMGP), and non-phosphorylated (dpMGP) [25], all these forms can be detected by ELISA, but only total MGP, rather than any specific form, could be detected by IHC. MGP was synthesized in bone and by many mesenchymal cells, and is highly expressed in vascular smooth muscle cells and chondrocytes [26]. Although the detailed mechanisms of action of MGP remain unclear, it is thought to be related to the inhibition of BMP-2 and BMP-4 by blocking calcium crystal deposition [27]. Abnormal physiological regulation of MGP has also been reported in association with vascular calcification and urinary calculi, and it was recently reported that MGP is thought to be associated with angiogenesis [28] and chemoresistance [29] in tumors. It was reported that MGP may alleviate inflammation in acute pancreatitis and arthritis in 2011, but it was rarely studied in recent years.

DH guinea pigs are characterized by development of a spontaneous knee OA that can be used to simulate the pathogenesis OA in the human knee and thus could be considered a useful animal study model in spontaneous OA [30]. In this study, we first confirmed the onset of spontaneous OA in the DH guinea pigs by histopathological examination, and we found that the degeneration of the knee cartilage after 6-months of age was significant in terms of higher MMS compared with younger subgroups and reference group. This finding is consistent with previous studies. For primary validation of the changes in MGP expression, we performed iTRAQ and confirmed that the MGP fraction was significantly greater in older (9- and 12-month-old) subgroups than in younger and reference groups. IHC provided further validation of these results by showing increasing MGP expression in cartilage with age, and MGP levels of > 6-month-old subgroups were significantly higher than those of younger and reference groups, and generally increased with month-ages. Therefore, the overall results of the various parts of our study are consistent.

Although some previous studies have reported that higher MGP levels are linked to elevated incidence and severity of OA, the results remained inconclusive [31]. It has been reported that high serum MGP levels were associated with the incidence of osteoarticular diseases and that the severity of nucleus pulposus degeneration was also reflected by MGP, but these conclusions were not confirmed in subsequent studies [31–33], and even an opposite conclusion was reported in a GWAS study [34] that found lower MGP expression linked to a higher incidence of hand OA. In this study, a useful animal model of spontaneous and progressive OA was utilized and verified, and the results again showed that increased expression of MGP in male DH guinea pigs was present throughout aging and disease progression and may be link to increased OA severity. Severe inflammation and osteophyte formation are manifestations of advanced OA. Although MGP has a protective effect, its protective effect is limited and does not strong enough to reverse inflammation and ossification. It also indicates that more evidence is needed to confirm these outcomes and fully understand the association between MGP and OA. Further studies should be planned.

Together with previous studies, the outcomes of this study may provide valid new evidence toward understanding the association between changes in MGP level and severity of OA. To the best of our knowledge, this is the first study investigating MGP levels and every stage of spontaneous OA in DH guinea pig model. A major methodological strength of this study was that a long-term and continuous observation on the animal model was conducted, especially for the early stage of spontaneous OA, which is seldom seen in the previous studies. Secondly, MGP levels were assessed as DEPs by iTRAQ and validated by IHC at every fixed interval. A correlation analysis between the measured MGP levels and OA severity at histopathologic MMS was also performed, and a significant positive link was detected, further indicating that MGP may be a potential marker of OA progression and worthy for further investigation.

There were also several important limitations in this study. First, limited by study conditions, guinea pig strains without spontaneous OA, such as strain-13 and BS-2 guinea pigs [12, 35], which would be considered ideal reference group against DH guinea pigs, could not to be obtained. We had to use the 1-month-old guinea pigs of the same DH strain as the reference group to minimize possible bias as much as possible. And, we had to use the background staining of 1-month-old guinea pigs as negative reference group. Secondly, because the details of the association between MGP and OA have not been clarified, and the role of MGP in OA progression or alleviation may be complex, the upstream and downstream genes to MGP should be directly identified or manipulated. Therefore, gene-gene interaction studies are also necessary for further study of MGP and OA. In addition, only IOC was used for the IHC analysis, limited by the amount of extracted protein, and Western Blot (WB) and Real-Time Quantitative Reverse Transcription PCR (qRT-PCR) were not performed for verification.

In this study based on spontaneous OA in the DH guinea pig model, we have described associations between MGP expression and the natural history of degenerative joint disease. As DH guinea pigs have similar spontaneous OA natural history with human, especially in histopathological manifestations of osteoarthritic knees [16], MGP may be worthy for future studied. Our results suggest that higher MGP levels may be present in association with progression of OA. Additional rigorously designed studies will be needed to further investigate the role of MGP in OA.

## Conclusion

Based on this study performed on male Dunkin-Hartley guinea pig spontaneous osteoarthritis model, our findings confirmed that elevated expression of MGP in DH guinea pigs may be concluded link to increased OA severity. Further studies are needed to investigate and confirm the association between MGP levels and OA severity.

## Supplementary Information

**Additional file 1.**

**Additional file 2.**

**Additional file 3.**

## Data Availability

All of the data in this study are obtained from experiments. The data used and analysed in this study are available from the corresponding author on reasonable request.

## Abbreviations

MGP: Matrix Gla (γ-carboxyglutamate) protein
OA: Osteoarthritis
DH guinea pig: Dunkin-Hartley guinea pig
MMS: Modified Mankin Score
IHC: Immunohistochemistry
iTRAQ: Isobaric tags for relative and absolute quantification
BMP-2/4: Bone morphogenetic protein 2/4
DEP: Differentially expressed protein
DAB: 3,3′-diaminobenzidine tetrahydrochloride
ANOVA: Analysis of variance
ICC: Intraclass correlation coefficient
OD: Optical density
WB: Western Blot
qRT-PCR: Real-Time Quantitative Reverse Transcription PCR
