# Supplementary material for: Defining matrix Gla protein expression in the Dunkin-Hartley guinea pig model of spontaneous osteoarthritis
Source: BMC Musculoskelet Disord. 2021 Oct 12;22:870. doi: 10.1186/s12891-021-04735-2 (PMC8513366; doi:10.1186/s12891-021-04735-2)
Supplement: Supplementary file 1 — Additional file 1. [file 12891_2021_4735_MOESM1_ESM.docx]

**Supplementary Table 1. Spearman correlation analysis of IOD-Trans and iTRAQs**

|  | IOD-Trans | iTRAQ-G1 | iTRAQ-G2 | iTRAQ-G3 |
| --- | --- | --- | --- | --- |
| IOD-Trans | 1 |  |  |  |
| iTRAQ-G1 | 0.8 | 1 |  |  |
| iTRAQ-G2 | 0.6 | 0.9* | 1 |  |
| iTRAQ-G3 | 0.6 | 0.9* | 1.000** | 1 |

* Correlation is significant at the 0.05 level (2-tailed).

** Correlation is significant at the 0.01 level (2-tailed).
